# The Survival Effect of Radiotherapy on Stage IIB/III Pancreatic Cancer Undergone Surgery in Different Age and Tumor Site Groups: A Propensity Scores Matching Analysis Based on SEER Database

**DOI:** 10.3389/fonc.2022.799930

**Published:** 2022-01-31

**Authors:** Dan Wang, Heming Ge, Mengxiang Tian, Chenglong Li, Lilan Zhao, Qian Pei, Fengbo Tan, Yuqiang Li, Chen Ling, Cenap Güngör

**Affiliations:** ^1^ Department of General Surgery, Xiangya Hospital, Central South University, Changsha, China; ^2^ National Clinical Research Center for Geriatric Disorders, Xiangya Hospital, Central South University, Changsha, China; ^3^ Department of General Visceral and Thoracic Surgery, University Medical Center Hamburg-Eppendorf, Hamburg, Germany; ^4^ Department of Thoracic Surgery, Fujian Provincial Hospital, Fuzhou, China

**Keywords:** pancreatic ductal adenocarcinoma, radiotherapy, SEER, Age, tumor site

## Abstract

**Background:**

It remains controversial whether radiotherapy (RT) improves survival in patients with stage IIB/III PDAC. A growing number of studies have found that patients’ age at diagnosis and tumor site not only affect prognosis, but also may lead to different treatment responses. Therefore, the purpose of this study was to verify whether the survival effect of radiotherapy in patients with stage IIB/III PDAC varies across age and tumor site groups.

**Methods:**

The target population was selected from PDAC patients undergone surgery in the Surveillance, Epidemiology, and End Results (SEER) database between 2004 and 2016. This study performed the Pearson’s chi-square test, Cox regression analysis, Kaplan-Meier (K-M) method, and focused on propensity frequency matching analysis.

**Results:**

Neither neoadjuvant radiotherapy (nRT) nor adjuvant radiotherapy (aRT) patient group had probably improved survival among early-onset patients. For middle-aged patients, nRT seemed to fail to extend overall survival (OS), while aRT might improve the OS. Plus, both nRT and aRT were associated with improved survival in elderly patients. The aRT might be related with survival benefits in patients with pancreatic head cancer, while nRT was not. And RT in patients with PDAC at other sites did not appear to provide a survival benefit.

**Conclusion:**

Carefully selected data from the SEER database suggested that age and tumor location may be the reference factors to guide the selection of RT for patients with stage IIB/III PDAC. These findings are likely to contribute to the development of personalized treatment for patients with stage IIB/III PDAC.

## Introduction

Pancreatic ductal adenocarcinoma (PDAC) is considered to be one of the most common gastrointestinal malignancies in the world, with an estimated incidence of 60,430 cases in 2021 (1). The prognosis of PDAC is dismal due to the characteristics of strong invasiveness and early metastasis. Even among PDAC patients with resectable disease, the 5-year overall survival (OS) rate is only 17% (2, 3). Therefore, in addition to the improvement of surgical methods, an increasing attention has been paid to the adjuvant treatment of pancreatic cancer, especially radiotherapy and chemotherapy. A large number of studies have shown that adjuvant chemotherapy can significantly improve the survival of PDAC patients (4). Accordingly, NCCN emphasizes the implementation of 6-months adjuvant chemotherapy for all PDAC patients undergoing surgical resection (5).

Radiotherapy (RT) is also one of the important weapons against PDAC, including neoadjuvant radiotherapy (nRT), adjuvant radiotherapy (aRT) and palliative treatment. It works by delivering ionizing radiation directly to the primary tumor and regional lymph nodes, which may cause genetic damage and ultimately apoptosis of cancer cells (6). However, our previous study has shown that RT does not benefit the survival of PDAC patients with stage T1-3N0M0 (7). For surgically resected PDAC patients, the NCCN and American Society for Radiation Oncology (ASTRO) also recommend conventional aRT for only a subset of high-risk patients (including positive lymph nodes (stage IIB/III) and margins) (5, 8). Although the role of RT as a local treatment in minimizing local recurrence has been widely recognized, there is no consensus on whether it can improve the survival of patients with stage IIB/III, when to use it, and how best to use it (9, 10).

In recent years, many studies have confirmed that the survival outcome and treatment effect of PDAC patients vary with age (11). Younger patients with PDAC tend to be at a more advanced stage and have a poorer prognosis than older patients, possibly due to their aggressive oncological behavior (12). In addition, younger PDAC patients are more likely to benefit from surgery and adjuvant chemotherapy compared with older patients, according to some studies (13, 14). However, there is still a lack of large sample studies on RT in PDAC patients of different ages. Also, the significance of primary tumor site for prognosis and treatment of patients with PDAC is still controversial. Among resected PDACs, those tumors located at the head of the pancreas had worse overall survival (OS) compared with those at the body and tail of the pancreas (15). Other studies have proved that tumor location does not affect the prognosis of PDAC, but has an important influence on postoperative recurrence and treatment methods (16).

Given the above questions, we used the Surveillance, Epidemiology, and End Results (SEER) database, which collects cancer case data from every state in the United States, to verify whether the survival effect of RT for stage IIB/III PDAC patients was different among different age and tumor site groups.

## Materials and Methods

### Data Extraction and Screening

**Figure 1:**
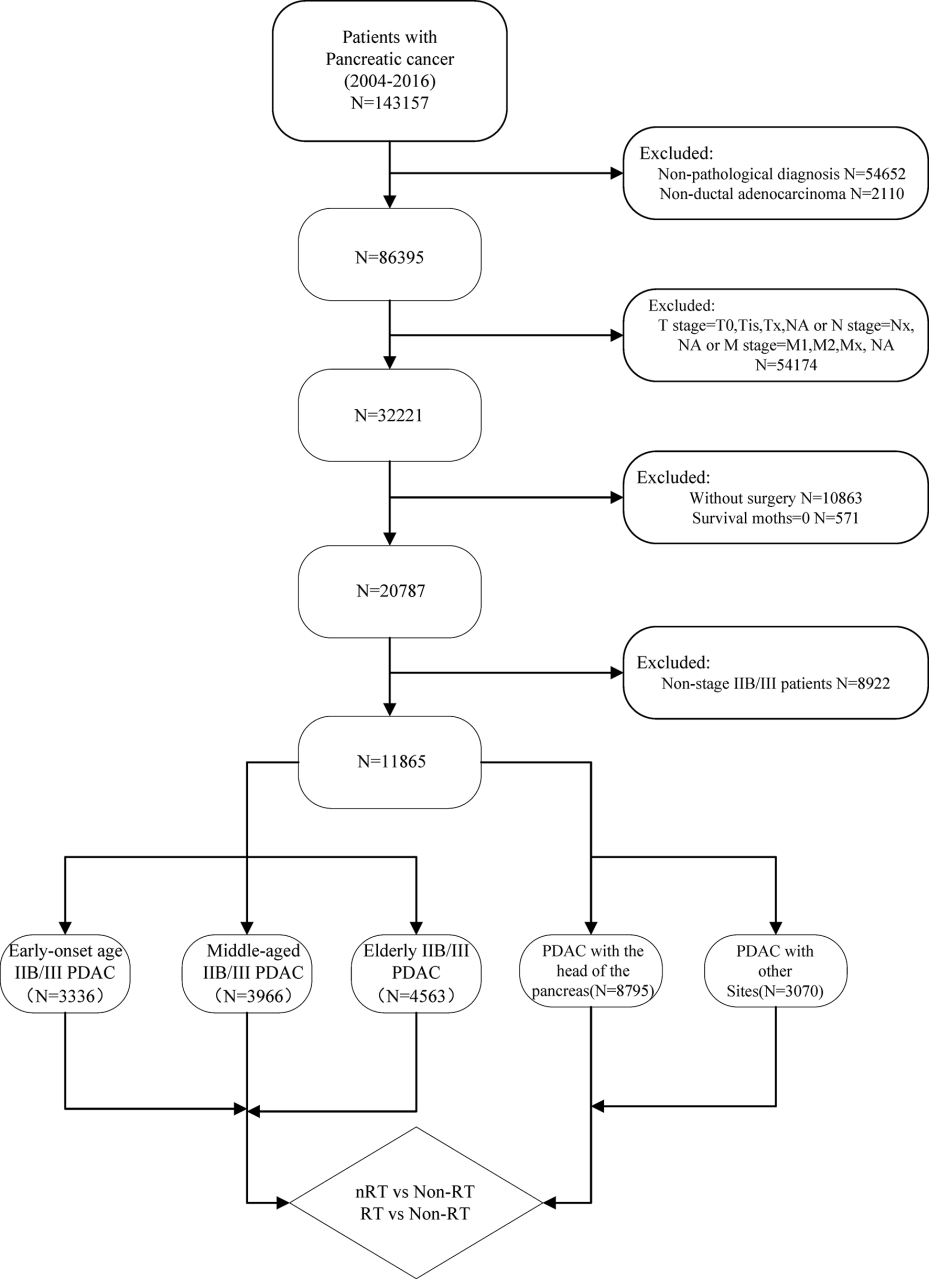
Procedures for inclusion and exclusion of PDAC patients.

Data for this retrospective study was collected from the SEER database (from 1973 to 2016), which includes 18 population-based cancer registries covering approximately 30% of the US population. The target population was limited to PDAC patients pathologically confirmed by post-operative specimens between 2004 and 2016. Other important information extracted included: general basic information, TNM stage, treatment information (surgery, chemotherapy, RT, Regional nodes examined (RNE)) and follow-up. In addition, the T and N stage were recorded according to the 8th edition TNM stage system by combining tumor size. Exclusion criteria were as follows: not confirmed by postoperative pathology (n=54,652), non-PDAC patients (n=2,110), non-stage IIB/III patients (n=54,174), non-surgical patients (n=10,863) and survival months is 0 (n = 571). Ultimately, enrolled patients were divided into three groups (age<60, 60-69 and ≥70) by the age at diagnosis and two cohorts (pancreatic head and other site groups) by the site of the primary tumor (**Figure 1**).

### Statistical Analysis

The endpoint of this study was overall survival (OS). Pearson’s chi-square test was used to analyze differences between groups. Multivariate Cox proportional risk regression model was performed to analysis the hazard ratio (HR) and 95% confidence interval (CI). The survival analysis was carried out by the Log-rank test, and the survival curve was drawn by Kaplan-Meier (K-M) method. We performed propensity score matching (PSM) to eliminate the influence of other variables. All statistical analyses in this study were conducted by software SPSS 25.0(IBM, Armonk, NY, USA). All p values less than 0.05 generated in the study were considered statistically significant.

## Results

### Characteristics and Survival Analysis of All Patients

The total population consisted of 11,865 PDAC patients with stage IIB/III, including 3,336 early-onset patients (age<60), 3,966 middle-aged patients (age: 60-69), and 4,563 elderly patients (age≥70). As shown in **Table 1**, there were significant differences in clinicopathological factors among these groups. The ratio of Grade III/IV was significantly higher in elderly patients with stage IIB/III PDAC compared to the other two subgroups (p<0.001). However, the proportion of T3 and T4 in early-onset patients was the highest among these three groups (p<0.001). What’s more, the age of patients with stage IIB/III PDAC appears to influence treatment selection and execution to some extent. Elderly patients with stage IIB/III PDAC were less likely to receive RT (27.13%), chemotherapy (60.31%), or surgery with RNE≥15 (52.33%) than early-onset (RT: 39.81%, chemotherapy: 72.54%, surgery with RNE≥15: 54.95%) and middle-aged patients (RT: 39.44%, chemotherapy: 74.13%, surgery with RNE≥ 15: 57.29%).

**Table 1:** Characteristics of stage IIB/III PDAC cancer.

Characteristics	Total	Age groups			p-value	Site groups		p-value
		Age < 60	60-69	Age≥70		Pancreas Head	Other sites	
Insurance					<0.001			0.010
Insured	9622 (81.10%)	2569 (77.01%)	3269 (82.43%)	3784 (82.93%)		7084 (80.55%)	2538 (82.67%)	
No/unknown	2243 (18.90%)	767 (22.99%)	697 (17.57%)	779 (17.07%)		1711 (19.45%)	532 (17.33%)	
Marital status					<0.001			0.500
Married	7521 (63.39%)	2092 (62.71%)	2648 (66.77%)	2781 (60.95%)		5564 (63.26%)	1957 (63.75%)	
Single	3957 (33.35%)	1123 (33.66%)	1193 (30.08%)	1641 (35.96%)		2952 (33.56%)	1005 (32.74%)	
Unknown	387 (3.26%%)	121 (3.63%)	125 (3.15%)	141 (3.09%)		279 (3.18%)	108 (3.51%)	
Race					<0.001			0.001
White	9820 (82.76%)	2618 (78.48%)	3294 (83.06%)	3908 (85.65%)		7337 (83.42%)	2483 (80.88%)	
Other	2045 (17.24%)	718 (21.52%)	672 (16.94%)	655 (14.35%)		1458 (16.58%)	587 (19.12%)	
Gender					<0.001			0.213
Male	6113 (51.52%)	1843 (55.25%)	2089 (52.67%)	2181 (47.80%)		4561 (51.86%)	1552 (50.55%)	
Female	5752 (48.48%)	1493 (44.75%)	1877 (47.33%)	2382 (52.20%)		4234 (48.14%)	1518 (49.45%)	
Tumor site					0.001			
Pancreas Head	8795 (74.13%)	2409 (72.21%)	3013 (75.97%)	3373 (73.92%)				
Pancreas Body Tail and other	3070 (25.87%)	927 (27.79%)	953 (24.03%)	1190 (26.08%)				
Grade					<0.001			<0.001
Grade I	1410 (11.88%)	491 (14.72%)	449 (11.32%)	470 (10.30%)		912 (10.37%)	498 (16.22%)	
Grade II	5366 (45.23%)	1445 (43.32%)	1806 (45.54%)	2115 (46.35%)		4039 (45.92%)	1327 (43.22%)	
Grade III/IV	4219 (35.56%)	1090 (32.67%)	1439 (36.28%)	1690 (37.04%)		3239 (36.83%)	980 (31.92%)	
Unknown	870 (7.33%)	310 (9.29%)	272 (6.86%)	288 (6.31%)		605 (6.88%)	265 (8.64%)	
T stage					<0.001			<0.001
T1	1393 (11.74%)	427 (12.80%)	446 (11.25%)	520 (11.40%)		1125 (12.79%)	268 (8.73%)	
T2	6484 (54.65%)	1728 (51.80%)	2167 (54.64%)	2589 (56.74%)		5250 (59.69%)	1234 (40.20%)	
T3	3018 (25.44%)	874 (26.20%)	1019 (25.69%)	1125 (24.65%)		1788 (20.33%)	1230 (40.07%)	
T4	970 (8.17%)	307 (9.20%)	334 (8.42%)	329 (7.21%)		632 (7.19%)	338 (11.00%)	
N stage					0.101			<0.001
N0	414 (3.49%)	127 (3.81%)	140 (3.53%)	147 (3.22%)		241 (2.74%)	173 (5.64%)	
N1	7336 (61.83%)	2029 (60.82%)	2418 (60.97%)	2889 (63.31%)		5262 (59.83%)	2074 (67.56%)	
N2	4115 (34.68%)	1180 (35.37%)	1408 (35.50%)	1527 (33.47%)		3292 (37.43%)	823 (26.80%)	
Radiation					<0.001			<0.001
Non- RT	7735 (65.19%)	2008 (60.19%)	2402 (60.56%)	3325 (72.87%)		5610 (63.79%)	2125 (69.22%)	
nRT	378 (3.19%)	137 (4.11%)	147 (3.71%)	94 (2.06%)		276 (3.14%)	102 (3.32%)	
RT	3752 (31.62%)	1191 (35.70%)	1417 (35.73%)	1144 (25.07%)		2909 (33.07%)	843 (27.46%)	
Chemotherapy					<0.001			<0.001
Yes	8112 (68.37%)	2420 (72.54%)	2940 (74.13%)	2752 (60.31%)		6232 (70.86%)	1880 (61.24%)	
No/Unknown	3753 (31.63%)	916 (27.46%)	1026 (25.87%)	1811 (39.69%)		2563 (29.14%)	1190 (38.76%)	
RNE					<0.001			<0.001
<15	5286 (44.55%)	1481 (44.39%)	1661 (41.88%)	2144 (46.99%)		3698 (42.05%)	1588 (51.73%)	
≥15	6493 (54.72%)	1833 (54.95%)	2272 (57.29%)	2388 (52.33%)		5034 (57.24%)	1459 (47.52%)	
Unknown	86 (0.73%)	22 (0.66%)	33 (0.83%)	31 (0.68%)		63 (0.71%)	23 (0.75%)	
Age								0.001
<60						2409 (27.39%)	927 (30.20%)	
60-69						3013 (34.26%)	953 (31.04%)	
≥70						3373 (38.35%)	1190 (38.76%)	

PDAC, Pancreatic Ductal Adenocarcinoma; RT, Radiotherapy; aRT, Adjuvant radiotherapy; nRT, Neoadjuvant radiotherapy.

In addition, 74.13% of primary tumors were located in the head of pancreas (8,795) and 25.87% in other sites (3,070) of the target population. Although the proportion of T3/T4 stages in patients with pancreatic head cancer is lower than tumors in other parts of the pancreas, more patients develop lymph node metastases. Similarly, patients with pancreatic head cancer tend to undergo RT (36.21%) and chemotherapy (70.86%) as well as surgery with RNE≥15 (57.24%) compared to patients with tumors in other sites of the pancreas (RT: 30.78%, chemotherapy: 61.24%, surgery with RNE≥ 15: 47.52%).

The results of univariate and multivariate cox proportional risk regression model (**Table 2**) indicated that the prognosis of all PDAC patients with stage IIB/III was closely related to insurance and marital status, gender, age at diagnosis, tumor location, tumor grade, tumor T and N stage, RT, chemotherapy and RNE (all P <0.001).

**Table 2:** Univariate and multivariate analysis for OS of all stage IIB/III PDAC patients.

Characteristics	Level	Univariate analysis	Multivariate analysis		
		P	HR	95%CI	P
Insurance Recode		<0.001			<0.001
	Insured		Reference	Reference	Reference
	No/unknown		1.150	1.091-1.211	<0.001
Marital status		<0.001			<0.001
	Married		Reference	Reference	Reference
	Single		1.122	1.071-1.176	<0.001
	Unknown		1.031	0.912-1.167	0.622
Age, years		<0.001			<0.001
	<60		Reference	Reference	Reference
	60-69		1.178	1.114-1.246	<0.001
	≥70		1.445	1.369-1.526	<0.001
Race recode		0.449			
	White				
	Other				
Sex		0.010			<0.001
	Female		Reference	Reference	Reference
	Male		1.102	1.054-1.151	<0.001
Tumor site		<0.001			<0.001
	Pancreas Head		Reference	Reference	Reference
	Other sites		0.850	0.806-0.895	<0.001
Grade		<0.001			<0.001
	I		Reference	Reference	Reference
	II		2.107	1.940-2.288	<0.001
	III/IV		2.762	2.540-3.004	<0.001
	Unknown		1.530	1.363-1.718	<0.001
T stage		<0.001			<0.001
	T1		Reference	Reference	Reference
	T2		1.296	1.207-1.391	<0.001
	T3		1.418	1.311-1.534	<0.001
	T4		2.008	1.788-2.256	<0.001
N stage		<0.001			<0.001
	N0		Reference	Reference	Reference
	N1		1.264	1.069-1.451	0.005
	N2		1.667	1.427-1.948	<0.001
Radiotherapy		<0.001			<0.001
	Non- RT		Reference	Reference	Reference
	nRT		0.910	0.792-1.045	0.181
	RT		0.891	0.848-0.937	<0.001
Chemotherapy		<0.001			<0.001
	Yes		Reference	Reference	Reference
	No		1.326	1.259-1.396	<0.001
RNE		<0.001			<0.001
	<15		Reference	Reference	Reference
	≥15		0.822	0.786-0.860	<0.001
	Unknown		1.183	0.938-1.491	0.155

PDAC, Pancreatic Ductal Adenocarcinoma; RT, Radiotherapy; aRT, Adjuvant radiotherapy; nRT, Neoadjuvant radiotherapy; OS, Overall Survival; CI, Confidence intervals; HR, Hazard ratios; RNE, Regional nodes examined.

### The Impact of RT on Early-Onset Patients With Stage IIB/III PDAC

**Figure 2:**
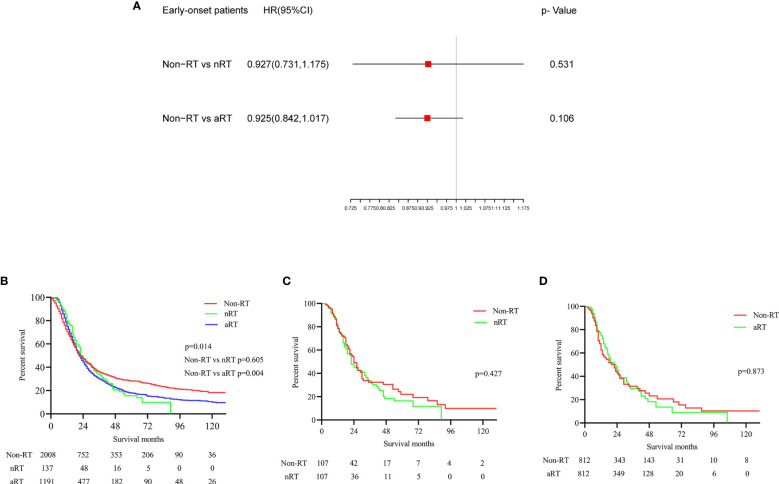
The forest plot and the survival curves were used to demonstrate the effect of radiotherapy in early onset stage IIB/III PDAC patients. **(A)**. The forest plot for non-RT vs. nRT and non-RT vs. aRT in early onset stage IIB/III PDAC patients; **(B)**. The survival curve for total early onset stage IIB/III PDAC patients before PSM; **(C)**. The survival curve for non-RT vs. nRT in early onset stage IIB/III PDAC patients after PSM; **(D)**. The survival curve for non-RT vs. aRT in early onset stage IIB/III PDAC patients after PSM.

According to the multivariate Cox regression model, neither nRT (p=0.531) nor aRT (p=0.106) improved OS in early-onset patients with stage IIB/III PDAC (**Figure 2A**). The K-M survival analysis showed that no significant association between nRT and OS (p=0.605), while aRT (p=0.004, HRs=1.132; 95% CIs, 1.038-1.235) developed worse OS compared to those with non-RT in early-onset patients (**Figure 2B**). The median survival of non-RT, nRT and aRT patients were 22, 22, and 21 months, respectively (**Table 3**). In order to reduce the interference of other variables, the balanced population of the non-RT and the nRT(n = 107 pairs), the non-RT and the RT(n = 812 pairs) were obtained by multiple 1:1 PSM for early-onset PDAC patients with stage IIB/III. Similarly, the survival curves after PSM indicated that nRT (p=0.427, **Figure 2C**) and aRT (p=0.873, **Figure 2D**) still did not seem to be associated with improved OS of early-onset patients with stage IIB/III PDAC.

**Table 3:** Median survival and, 1-, 3- and 5-year OS of stage IIB/III PDAC patients.

Groups	Treatments	Median survival	1-year OS	3-year OS	5-year OS
Age < 60	Non-RT	22	47.17%	26.01%	19.08%
	nRT	22	47.92%	9.33%	–
	RT	21	44.75%	16.33%	10.08%
60-69	Non-RT	18	38.83%	13.83%	9.67%
	nRT	21	45.50%	12.49%	6.58%
	RT	23	44.08%	13.85%	10.67%
Age≥70	Non-RT	13	29.17%	7.90%	3.75%
	nRT	23	47.17%	14.25%	10.41%
	RT	19	39.92%	10.08%	3.81%
Pancreas Head	Non-RT	16	32.25%	11.08%	7.58%
	nRT	20	47.16%	12.16%	–
	RT	22	43.42%	13.17%	8.33%
Other sites	Non-RT	20	45.08%	22.92%	15.91%
	nRT	24	52.75%	9.01%	7.25%
	RT	20	39.66%	12.58%	9.08%

PDAC, Pancreatic Ductal Adenocarcinoma; RT, Radiotherapy; aRT, Adjuvant radiotherapy; nRT, Neoadjuvant radiotherapy; OS, Overall Survival.

### The Impact of RT on Middle-Aged Patients With Stage IIB/III PDAC

**Figure 3:**
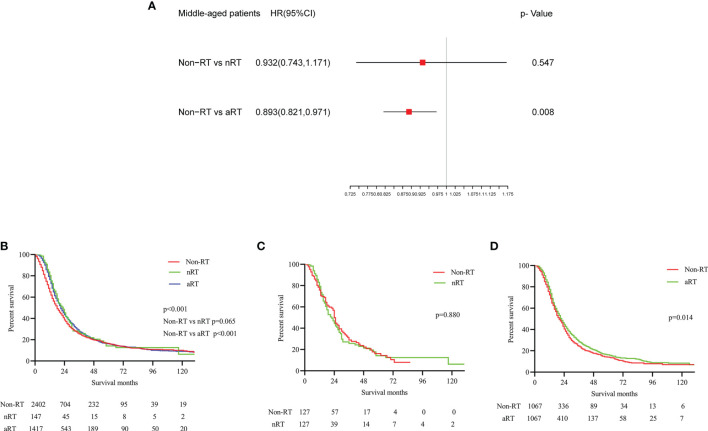
The forest plot and the survival curves were used to demonstrate the effect of radiotherapy in middle-aged stage IIB/III PDAC patients. **(A)**. The forest plot for non-RT vs. nRT and non-RT vs. aRT in middle-aged stage IIB/III PDAC patients; **(B)**. The survival curve for total middle-aged stage IIB/III PDAC patients before PSM; **(C)**. The survival curve for non-RT vs. nRT in middle-aged stage IIB/III PDAC patients after PSM; **(D)**. The survival curve for non-RT vs. aRT in middle-aged stage IIB/III PDAC patients after PSM.

Using similar methods, the multivariate Cox regression analysis and K-M survival analysis before matching showed that nRT seemed to fail to prolong the OS of middle-aged patients with stage IIB/III PDAC (p=0.547**;** p=0.065), while aRT might improve the OS of patients (p=0.008, **Figure 3A**; p<0.001, HRs=0.869; 95% CIs, 0.806-0.938, **Figure 3B**). Median survival was 18, 21 and 23 months for patients receiving non-RT, nRT and aRT, respectively. The balanced populations of non-RT and nRT (n = 127 pairs), non-RT and aRT (n = 1067 pairs) were matched by 1:1 PSM. Further survival analysis found that nRT could not improve the OS (p=0.880, **Figure 3C**), and the OS of aRT was significantly better than that of non-RT in middle-aged PDAC patients with stage IIB/III (p=0.014, HRs=0.883; 95% CIs, 0.798-0.977, **Figure 3D**).

### The Impact of RT on Elderly Patients With Stage IIB/III PDAC

**Figure 4:**
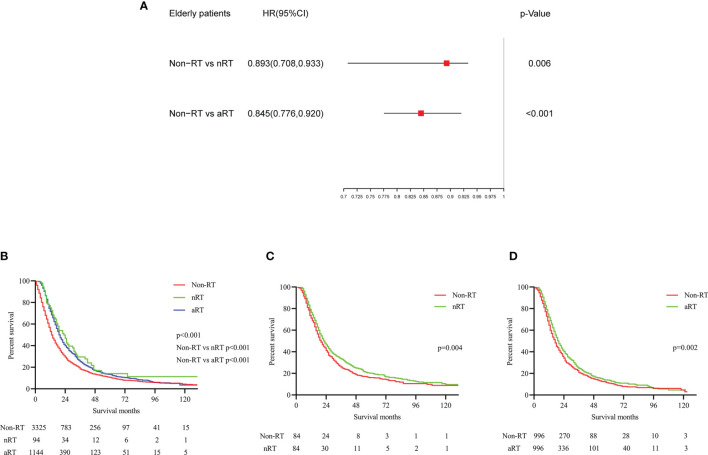
The forest plot and the survival curves were used to demonstrate the effect of radiotherapy in elderly stage IIB/III PDAC patients. **(A)**. The forest plot for non-RT vs. nRT and non-RT vs. aRT in elderly stage IIB/III PDAC patients; **(B)**. The survival curve for total elderly stage IIB/III PDAC patients before PSM; **(C)**. The survival curve for non-RT vs. nRT in elderly stage IIB/III PDAC patients after PSM; **(D)**. The survival curve for non-RT vs. aRT in elderly stage IIB/III PDAC patients after PSM.

Before PSM matching, both Cox multivariate regression analysis (**Figure 4A**) and K-M (**Figure 4B**) survival analysis showed that nRT and aRT might be related with survival benefits for elderly patients. The median survival for these three treatments were 13 (non-RT), 23(nRT) and 19 months(aRT), respectively. Similarly, the K-M survival analysis after matching suggested that nRT(p=0.004, HRs=0.848; 95% CIs, 0.755-0.953, **Figure 4C**) and aRT(p=0.002, HRs=0.853; 95% CIs, 0.770-0.944, **Figure 4D**) still seemed to provided significant survival benefits for elderly PDAC patients with stage IIB/III.

### The Impact of RT on Stage IIB/III PDAC Patients With Different Tumor Sites

**Figure 5:**
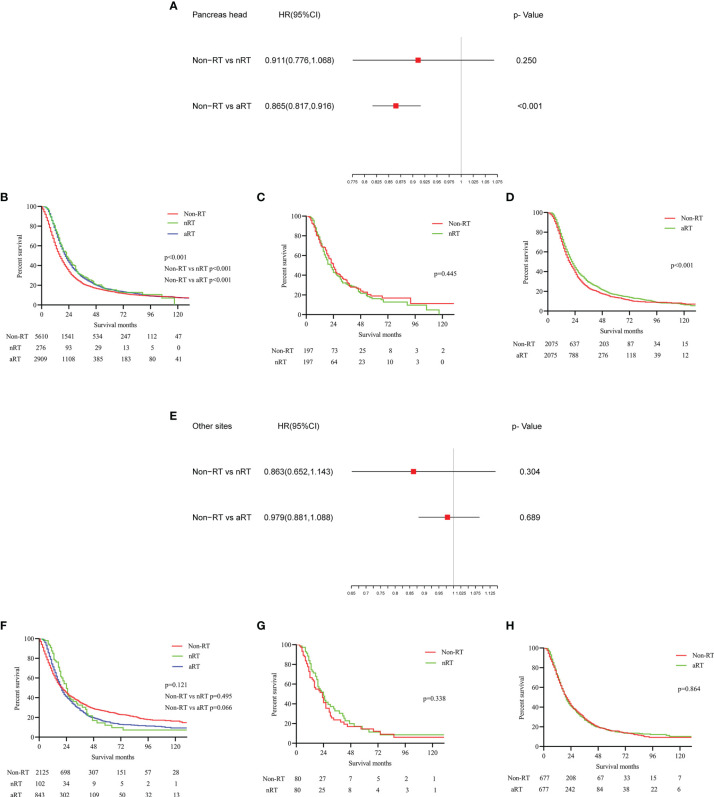
The forest plots and the survival curves were used to demonstrate the effect of radiotherapy in stage IIB/III PDAC patients with different tumor sites. **(A)** The forest plot for non-RT vs. nRT and non-RT vs. aRT in patients with pancreatic head tumors; **(B)** The survival curve for total patients with pancreatic head tumors before PSM; **(C)** The survival curve for non-RT vs. nRT in patients with pancreatic head tumors after PSM; **(D)** The survival curve for non-RT vs. aRT in patients with pancreatic head tumors after PSM; **(E)** The forest plot for non-RT vs. nRT and non-RT vs. aRT in PDAC patients at other sites; **(F)** The survival curve for total PDAC patients at other sites before PSM; **(G)** The survival curve for non-RT vs. nRT in PDAC patients at other sites after PSM; **(H)** The survival curve for non-RT vs. aRT in PDAC patients at other sites after PSM.

The same approaches were used to analyze patients with stage IIB/III PDAC at different sites. According to Cox multivariate regression analysis of pancreatic head cancer, there was no significant difference in OS between nRT patients and non-RT patients(p=0.250), while the OS of aRT patients was significantly better than that of non-RT patients (p<0.001, **Figure 5A**). The K-M survival curves suggested that both nRT and aRT were beneficial for OS in patients with pancreatic head cancer before PSM (all p<0.001, **Figure 5B**). The corresponding median survival were 16(non-RT), 20(nRT), and 22(aRT) months, respectively. The survival curve after PSM showed that although there was no significant difference in survival between the nRT and non-RT groups (p=0.445, **Figure 5C**), aRT still improved the OS of patients with pancreatic head cancer (p<0.001, HRs=0.867; 95% CIs, 0.807-0.932, **Figure 5D**).

For patients with stage IIB/III PDAC at other sites, the multivariate Cox regression analysis (**Figure 5E**) and K-M survival analysis without PSM (**Figure 5F**) showed that neither aRT nor nRT were associated with improved survival, which was further validated by survival analysis with PSM (**Figures 5G, H**). Median survival was 20, 24 and 20 months for patients undergone non-RT, nRT and aRT, respectively.

## Discussion

A variety of tumors, including pancreatic cancer, possess different molecular characteristics, biological behaviors and therapeutic responses in different age groups (17–19). For example, young patients and elderly patients with pancreatic cancer benefit from comprehensive treatment differently. Another study found that chemotherapy didn’t seem to affect the prognosis of young patients with breast cancer, which is obviously inconsistent with most studies that are not grouped by age (20). In addition, a recent study focusing on stage II/III rectal cancer revealed that radiotherapy had different effects on the survival of patients at different ages (21). Moreover, treatment methods and postoperative recurrence methods are also diverse according to the different site of primary tumor of PDAC (16). These evidences prompted us to explore the impact of radiotherapy on survival in PDAC patients with stage IIB/III at different ages and sites through the SEER database. The results of our study showed that the survival effect of RT was not consistent in different age groups, but also in different tumor sites.

We found that RT failed to benefit the survival of patients with early-onset stage IIB/III PDAC through age stratification. Even before PSM, survival analysis indicated that aRT was a risk factor for prognosis, which was clearly at odds with the findings of most studies that do not group by age (22). Conventional wisdom has it that younger patients are more likely to withstand more aggressive treatments, because of their relatively good physical state (23). The data we selected also demonstrated that patients with early-onset PDAC underwent more extensive surgery (RNE≥15) and chemoradiotherapy than older patients. Better treatment utilization and the ability to tolerate intensive therapy will hopefully be associated with improved outcomes. However, our data do not support that increasing RT in early-onset patients improves prognosis. A retrospective study from the Ellis Fisher Cancer Center also found that younger pancreatic cancer patients who received more treatment did not have a greater survival benefit than older patients (24). This suggested that survival improvements in early-onset patients with stage IIB/III PDAC are more likely to depend on the development of new therapies and technologies, rather than more aggressive use of existing models.

Traditionally, RT has been considered to cause significant radiation toxicity to PDAC due to the presence of many radiation-sensitive organs (stomach, duodenum, liver, kidney and spinal cord) in the pancreatic anatomic region (25). Furthermore, the conventional wisdom has been that increasing age, comorbidities, and worsening physical conditions (such as frailty) increase the risk of chemotherapy intolerance, disease progression, and death (26). Therefore, the application of RT in elderly patients with PDAC is more cautious in real clinical practice. However, recent studies have found that age may not be a predictor of radiation-induced toxicity and that healthy status such as frailty are more closely associated with radiation toxicity. Frailty is a pathological condition characterized by the decline of various physiological systems, although related to age, but not equal to old age (27). In this study, the proportion of patients over 70 years old who received RT was significantly lower than that of patients with early onset, especially the ration of nRT was only 2.06%. However, survival analysis showed that aRT could prolong survival in middle-aged and elderly patients, and nRT improved survival in the elderly. It is necessary for us to re-evaluate the benefits and risks of RT in elderly PDAC patients. In fact, many analyses showed that in terms of radiotherapy tolerance and toxicity, the results of elderly patients were similar to those of the general population, including young patients (28, 29). In adjuvant therapy, radiotherapy and chemotherapy are carried out at the same time, which can eradicate residual microscopic or macroscopic disease caused by the special anatomy of the pancreatic lesion (22). Moreover, compared with aRT, nRT is associated with a significant reduction in local recurrence and treatment-related toxicity (9). Therefore, clinicians should pay attention to the use of aRT in patients over 60 years of age with stage IIB/III PDAC and nRT in patients over 70 years of age.

In addition, the effect of tumor anatomical site on the prognosis and treatment of pancreatic cancer has gradually become a research hotspot in recent years. A retrospective study of 128 patients with pancreatic cancer from Japan showed that tumor location was not a prognostic factor for overall survival of locally advanced pancreatic cancer, although the clinical presentation of PDAC at different sites may differ (30). However, pancreatic head tumors had a higher rate of lymph node metastasis and a correspondingly poorer prognosis according to another propensity score-matched analysis (31). Another study, which analyzed germline and somatic mutations in 90 Chinese patients with pancreatic cancer, found differences in the mutation spectrum of pancreatic tumors at different anatomic sites, suggesting that treatment options for patients at different tumor sites may differ (32). In our experience, pancreatic head cancer had a worse prognosis than tumors in other sites. What’s more, there were differences in the effects of RT in patients with stage IIB/III PDAC at different sites. The application of aRT can benefit the OS in patients with pancreatic head cancer, but not in patients with PDAC at other sites. Therefore, we suggested clinicians should also consider tumor site as an important factor in deciding radiotherapy options for pancreatic cancer.

To date, our study was the first to specifically investigate the impact of RT on survival in PDAC patients with stage IIB/III at different ages and tumor sites. As a retrospective, non-randomized study, selection bias and confounding factors inevitably interfered. Although we use PSM to try to compensate for these defects, there are still some confounding factors that cannot be identified and some known confounding factors that cannot be controlled. The degree of tumor invasion, health status (comorbidities and frailty), surgical complications and recovery are all important factors affecting the decision of radiotherapy. These variables cannot be obtained and coded directly in the SEER database, so they can only be controlled indirectly. Furthermore, the data was sourced from a public database (SEER) rather than a separate queue, and the available information was limited. For example, the SEER database does not provide ECOG performance status, resectability status, surgical margin status, radiotherapy target design, technique, and dose, which undoubtedly weakens the reliability of the conclusions of this study. In addition, the data only provided whether the patients underwent chemotherapy, so it was not possible to determine whether the patients underwent 5-fluorouracil-based regimen. Finally, genomic data from tumor samples also have great clinical reference value to guide prognosis and treatment, but this is also not recorded in the SEER database. These missing variables are critical to prognosis and need to be discussed in future studies.

In summary, this study was based on a stratified analysis of age and tumor location, highlighting the difference in the efficacy of RT in different subgroups of patients. Of course, the results of this study need to be further confirmed by prospective cohorts in patients with stage IIB/III PDAC.

## Conclusion

Carefully selected data from the SEER database suggested that age and tumor location may be the reference factors to guide the selection of RT for patients with stage IIB/III PDAC. These findings may contribute to the development of individualized treatment for patients with stage IIB/III PDAC.

## Data Availability Statement

Publicly available datasets were analyzed in this study. This data can be found here: https://seer.cancer.gov/.

## Ethics Statement

The studies involving human participants were reviewed and approved by Ethics Committee of Xiangya Hospital of Central South University. Written informed consent from the participants’ legal guardian/next of kin was not required to participate in this study in accordance with the national legislation and the institutional requirements.

## Author Contributions

Conceptualization: DW, CL, and CG. Data curation: HG, MT, and CLL. Formal analysis: DW, LZ, QP, and FT. Methodology: DW, YL, QP, and FT. Writing-original draft: DW. Writing review & editing: CL. Revise & proofread: DW, YL, CG, and CL. All the authors read and approved the final manuscript.

## Funding

The first author, DW, received funding from the China Scholarship Council. The corresponding author, CL, received a grant from the Nature Scientific Foundation of China, contract grant number: 8217131506.

## Conflict of Interest

The authors declare that the research was conducted in the absence of any commercial or financial relationships that could be construed as a potential conflict of interest.

## Publisher’s Note

All claims expressed in this article are solely those of the authors and do not necessarily represent those of their affiliated organizations, or those of the publisher, the editors and the reviewers. Any product that may be evaluated in this article, or claim that may be made by its manufacturer, is not guaranteed or endorsed by the publisher.

## Abbreviations

PDAC, Pancreatic Ductal Adenocarcinoma; RT, Radiotherapy; aRT, Adjuvant radiotherapy; nRT, Neoadjuvant radiotherapy; ASTRO, American Society for Radiation Oncology; SEER, Surveillance, Epidemiology, and End Results; OS, Overall Survival; CIs, Confidence intervals; HRs, Hazard ratios; US, United States; PSM, Propensity score matching; RNE, Regional nodes examined.
