# Supplementary material for: The Survival Effect of Radiotherapy on Stage IIB/III Pancreatic Cancer Undergone Surgery in Different Age and Tumor Site Groups: A Propensity Scores Matching Analysis Based on SEER Database
Source: Front Oncol. 2022 Jan 31;12:799930. doi: 10.3389/fonc.2022.799930 (PMC8841859; doi:10.3389/fonc.2022.799930)
Supplement: Supplementary file 1 [file Table_1.docx]

Supplementary Table 1. Features of early-onset patients in the non-radiotherapy group and the neoadjuvant radiotherapy group before and after PSM.

| Characteristics | Before PSM | | |  | After PSM | | |
| --- | --- | --- | --- | --- | --- | --- | --- |
|  | Non-radiotherapy | Neoadjuvant radiotherapy | P |  | Non-radiotherapy | Neoadjuvant radiotherapy | P |
| Insurance Recode |  |  | 0.743 |  |  |  | 1.000 |
| Insured | 1595(79.43%) | 111(81.02%) |  |  | 86(80.37%) | 87(81.31%) |  |
| No/unknown | 413(20.57%) | 26(18.98%) |  |  | 21(19.63%) | 20(18.69%) |  |
| Marital status |  |  | 0.017 |  |  |  | 0.374 |
| Married | 1219(60.71%) | 99(72.26%) |  |  | 74(69.16%) | 80(74.77%) |  |
| Single | 709(35.31%) | 32(23.36%) |  |  | 30(28.04%) | 22(20.56%) |  |
| Unknown | 80(3.98%) | 6(4.38%) |  |  | 3(2.80%) | 5(4.67%) |  |
| Race |  |  | <0.001 |  |  |  | 1.000 |
| White | 1546(76.99%) | 123(89.78%) |  |  | 95(88.79%) | 94(87.85%) |  |
| Others | 462(23.01%) | 14(10.22%) |  |  | 12(11.21%) | 13(12.15%) |  |
| Sex |  |  | 0.307 |  |  |  | 0.891 |
| Male | 892(44.42%) | 67(48.91%) |  |  | 49(45.79%) | 51(47.66%) |  |
| Female | 1116(55.58%) | 70(51.09%) |  |  | 58(54.21%) | 56(52.34%) |  |
| Tumor site |  |  | 0.126 |  |  |  | 0.637 |
| Pancreas Head | 1377(68.58%) | 103(75.18%) |  |  | 78(72.90%) | 82(76.64%) |  |
| Pancreas Body Tail and other | 631(31.42%) | 34(24.82%) |  |  | 29(27.10%) | 25(23.36%) |  |
| Grade |  |  | <0.001 |  |  |  | 0.346 |
| I | 376(18.73%) | 7(5.10%) |  |  | 3(2.80%) | 7(6.54%) |  |
| II | 821(40.89%) | 44(32.12%) |  |  | 38(35.51%) | 36(33.64%) |  |
| III/IV | 617(30.73%) | 32(23.36%) |  |  | 31(28.97%) | 23(21.50%) |  |
| Unknown | 194(9.65%) | 54(39.42%) |  |  | 35(32.72%) | 41(38.32%) |  |
| T stage |  |  | <0.001 |  |  |  | 0.422 |
| T1 | 252(12.55%) | 6(4.38%) |  |  | 6(5.61%) | 6(5.61%) |  |
| T2 | 1051(52.34%) | 47(34.30%) |  |  | 44(41.12%) | 47(43.92%) |  |
| T3 | 559(27.84%) | 22(16.06%) |  |  | 32(29.91%) | 22(20.56%) |  |
| T4 | 146(7.27%) | 62(45.26%) |  |  | 25(23.36%) | 32(29.91%) |  |
| N stage |  |  | <0.001 |  |  |  | 0.653 |
| N0 | 54(2.69%) | 46(33.58%) |  |  | 16(14.95%) | 19(17.76%) |  |
| N1 | 1208(60.16%) | 77(56.20%) |  |  | 80(74.77%) | 74(69.16%) |  |
| N2 | 746(37.15%) | 14(10.22%) |  |  | 11(10.28%) | 14(13.08%) |  |
| Chemotherapy |  |  | <0.001 |  |  |  | 1.000 |
| Yes | 1142(56.87%) | 137(100%) |  |  | 107(100%) | 107(100%) |  |
| No/Unknown | 866(43.13%) | 0 |  |  | 0 | 0 |  |
| RNE |  |  | 0.003 |  |  |  | 0.359 |
| <15 | 891(44.37%) | 75 (54.74%) |  |  | 48(44.86%) | 57(53.27%) |  |
| ≥15 | 1106(55.08%) | 59(43.07%) |  |  | 58(54.21%) | 48(44.86%) |  |
| Unknown | 11(0.55%) | 3(2.19%) |  |  | 1(0.93%) | 2(1.87%) |  |

Abbreviations PSM: Propensity score matching; RNE: Regional nodes examined
