# Supplementary material for: The Survival Effect of Radiotherapy on Stage IIB/III Pancreatic Cancer Undergone Surgery in Different Age and Tumor Site Groups: A Propensity Scores Matching Analysis Based on SEER Database
Source: Front Oncol. 2022 Jan 31;12:799930. doi: 10.3389/fonc.2022.799930 (PMC8841859; doi:10.3389/fonc.2022.799930)
Supplement: Supplementary file 2 [file Table_2.docx]

Supplementary Table 2. Features of early-onset patients in the non-radiotherapy group and the adjuvant radiotherapy group before and after PSM.

| Characteristics | Before PSM | | |  | After PSM | | |
| --- | --- | --- | --- | --- | --- | --- | --- |
|  | Non-radiotherapy | Adjuvant radiotherapy | P |  | Non-radiotherapy | Adjuvant radiotherapy | P |
| Insurance Recode |  |  | <0.001 |  |  |  | 1.000 |
| Insured | 1595(79.43%) | 863(72.46%) |  |  | 667(82.14%) | 667(82.14%) |  |
| No/unknown | 413(20.57%) | 328(27.54%) |  |  | 145(17.86%) | 145(17.86%) |  |
| Marital status |  |  | 0.034 |  |  |  | 0.966 |
| Married | 1219(60.71%) | 774(64.99%) |  |  | 537(66.13%) | 537(66.13%) |  |
| Single | 709(35.31%) | 382(32.07%) |  |  | 268(33.00%) | 267(32.88%) |  |
| Unknown | 80(3.98%) | 35(2.94%) |  |  | 7(0.87%) | 8(0.99%) |  |
| Race |  |  | 0.076 |  |  |  | 0.715 |
| White | 1546(76.99%) | 949(79.68%) |  |  | 644(79.31%) | 638(78.57%) |  |
| Others | 462(23.01%) | 242(20.32%) |  |  | 168(20.69%) | 174(21.43%) |  |
| Sex |  |  | 0.820 |  |  |  | 0.960 |
| Male | 892(44.42%) | 534(44.84%) |  |  | 373(45.94%) | 372(45.81%) |  |
| Female | 1116(55.58%) | 657(55.16%) |  |  | 439 (54.06%) | 440(54.19%) |  |
| Tumor site |  |  | <0.001 |  |  |  | 0.950 |
| Pancreas Head | 1377(68.58%) | 929(78.00%) |  |  | 650(80.05%) | 651(80.17%) |  |
| Pancreas Body Tail and other | 631(31.42%) | 262(22.00%) |  |  | 162(19.95%) | 161(19.83%) |  |
| Grade |  |  | <0.001 |  |  |  | 1.000 |
| I | 376(18.73%) | 108(9.07%) |  |  | 61(7.51%) | 61(7.51%) |  |
| II | 821(40.89%) | 580(48.70%) |  |  | 407(50.12%) | 407(50.12%) |  |
| III/IV | 617(30.73%) | 441(37.03%) |  |  | 317(39.04%) | 317(39.04%) |  |
| Unknown | 194(9.65%) | 62(5.20%) |  |  | 27(3.33%) | 27(3.33%) |  |
| T stage |  |  | 0.134 |  |  |  | 0.999 |
| T1 | 252(12.55%) | 169(14.19%) |  |  | 95(11.70%) | 96(11.82%) |  |
| T2 | 1051(52.34%) | 630(52.90%) |  |  | 496(61.08%) | 496(61.08%) |  |
| T3 | 559(27.84%) | 293(24.60%) |  |  | 195(24.02%) | 195(24.02%) |  |
| T4 | 146(7.27%) | 99(8.31%) |  |  | 26(3.20%) | 25(3.08%) |  |
| N stage |  |  | 0.385 |  |  |  | 1.000 |
| N0 | 54(2.69%) | 27(2.27%) |  |  | 4(0.49%) | 4(0.49%) |  |
| N1 | 1208(60.16%) | 744(62.47%) |  |  | 497(61.21%) | 497(61.21%) |  |
| N2 | 746(37.15%) | 420(35.26%) |  |  | 311(38.30%) | 311(38.30%) |  |
| Chemotherapy |  |  | <0.001 |  |  |  | 1.000 |
| Yes | 1142(56.87%) | 1141(95.80%) |  |  | 780(96.06%) | 780(96.06%) |  |
| No/Unknown | 866(43.13%) | 50(4.20%) |  |  | 32(3.94%) | 32(3.94%) |  |
| RNE |  |  | 0.760 |  |  |  | 0.459 |
| <15 | 891(44.37%) | 515(43.24%) |  |  | 339(41.75%) | 317(39.04%) |  |
| ≥15 | 1106(55.08%) | 668(56.09%) |  |  | 469(57.76%) | 489(60.22%) |  |
| Unknown | 11(0.55%) | 8(0.67%) |  |  | 4(0.49%) | 6(0.74%) |  |

Abbreviations PSM: Propensity score matching; RNE: Regional nodes examined
