# Supplementary material for: The Survival Effect of Radiotherapy on Stage IIB/III Pancreatic Cancer Undergone Surgery in Different Age and Tumor Site Groups: A Propensity Scores Matching Analysis Based on SEER Database
Source: Front Oncol. 2022 Jan 31;12:799930. doi: 10.3389/fonc.2022.799930 (PMC8841859; doi:10.3389/fonc.2022.799930)
Supplement: Supplementary file 3 [file Table_3.docx]

Supplementary Table 3. Features of middle-aged patients in the non-radiotherapy group and the neoadjuvant radiotherapy group before and after PSM.

| Characteristics | Before PSM | | |  | After PSM | | |
| --- | --- | --- | --- | --- | --- | --- | --- |
|  | Non-radiotherapy | Neoadjuvant radiotherapy | P |  | Non-radiotherapy | Neoadjuvant radiotherapy | P |
| Insurance Recode |  |  | 0.091 |  |  |  | 0.145 |
| Insured | 2045(85.14%) | 133(90.48%) |  |  | 105(82.68%) | 114(89.76%) |  |
| No/unknown | 357(14.86%) | 14(9.52%) |  |  | 22(17.32%) | 13(10.24%) |  |
| Marital status |  |  | 0.013 |  |  |  | 0.399 |
| Married | 1558(64.86%) | 112(76.19%) |  |  | 98(77.17%) | 96(75.59%) |  |
| Single | 769(32.02%) | 30(20.41%) |  |  | 28(22.05%) | 27(21.26%) |  |
| Unknown | 75(3.12%) | 5(3.40%) |  |  | 1(0.78%) | 4(3.15%) |  |
| Race |  |  | 0.649 |  |  |  | 0.733 |
| White | 1995(83.06%) | 125(85.03%) |  |  | 105(82.68%) | 108(85.04%) |  |
| Others | 407(16.94%) | 22(14.97%) |  |  | 22(17.32%) | 19(14.96%) |  |
| Sex |  |  | 0.335 |  |  |  | 0.900 |
| Male | 1144(47.63%) | 64(43.54%) |  |  | 57(44.88%) | 56(44.09%) |  |
| Female | 1258(52.37%) | 83(56.46%) |  |  | 70(55.12%) | 71(55.91%) |  |
| Tumor site |  |  | 0.496 |  |  |  | 0.776 |
| Pancreas Head | 1792(74.60%) | 106(72.11%) |  |  | 92(72.44%) | 95(74.80%) |  |
| Pancreas Body Tail and other | 610(25.40%) | 41(27.89%) |  |  | 35(27.56%) | 32(25.20%) |  |
| Grade |  |  | <0.001 |  |  |  | 0.053 |
| I | 300(12.49%) | 18(12.24%) |  |  | 4(3.15%) | 8(6.30%) |  |
| II | 1044(43.46%) | 47(31.97%) |  |  | 52(40.94%) | 47(37.01%) |  |
| III/IV | 903(37.59%) | 34(23.14%) |  |  | 48(37.80%) | 34(26.77%) |  |
| Unknown | 155(6.46%) | 48(32.65%) |  |  | 23(18.11%) | 38(29.92%) |  |
| T stage |  |  | <0.001 |  |  |  | 0.429 |
| T1 | 288(11.99%) | 2(1.36%) |  |  | 3(2.36%) | 2(1.57%) |  |
| T2 | 1284(53.46%) | 54(36.73%) |  |  | 54(42.51%) | 54(42.52%) |  |
| T3 | 659(27.44%) | 32(21.77%) |  |  | 40(31.50%) | 31(24.41%) |  |
| T4 | 171(7.11%) | 59(40.14%) |  |  | 30(23.63%) | 40(31.50%) |  |
| N stage |  |  | <0.001 |  |  |  | 0.981 |
| N0 | 66(2.75%) | 42(28.57%) |  |  | 25(19.69%) | 25(19.69%) |  |
| N1 | 1459(60.74%) | 89(60.54%) |  |  | 87(68.50%) | 86(67.72%) |  |
| N2 | 877(36.51%) | 16(10.88%) |  |  | 15(11.81%) | 16(12.59%) |  |
| Chemotherapy |  |  | <0.001 |  |  |  | 1.000 |
| Yes | 1456(60.62%) | 145(98.64%) |  |  | 126(99.21%) | 125(98.43%) |  |
| No/Unknown | 946(39.38%) | 2(1.36%) |  |  | 1(0.79%) | 2(1.57%) |  |
| RNE |  |  | <0.001 |  |  |  | 0.508 |
| <15 | 955(39.76%) | 77(52.38%) |  |  | 66(51.97%) | 63(49.61%) |  |
| ≥15 | 1429(59.49%) | 64(43.54%) |  |  | 59(46.46%) | 59(46.46%) |  |
| Unknown | 18(0.75%) | 6(4.08%) |  |  | 2(1.57%) | 5(3.93%) |  |

Abbreviations PSM: Propensity score matching; RNE: Regional nodes examined
