# Supplementary material for: The Survival Effect of Radiotherapy on Stage IIB/III Pancreatic Cancer Undergone Surgery in Different Age and Tumor Site Groups: A Propensity Scores Matching Analysis Based on SEER Database
Source: Front Oncol. 2022 Jan 31;12:799930. doi: 10.3389/fonc.2022.799930 (PMC8841859; doi:10.3389/fonc.2022.799930)
Supplement: Supplementary file 4 [file Table_4.docx]

Supplementary Table 4. Features of middle-aged patients in the non-radiotherapy group and the adjuvant radiotherapy group before and after PSM.

| Characteristics | Before PSM | | |  | After PSM | | |
| --- | --- | --- | --- | --- | --- | --- | --- |
|  | Non-radiotherapy | Adjuvant radiotherapy | P |  | Non-radiotherapy | Adjuvant radiotherapy | P |
| Insurance Recode |  |  | <0.001 |  |  |  | 0.841 |
| Insured | 2045(85.14%) | 1091(76.99%) |  |  | 941(88.19%) | 938(87.91%) |  |
| No/unknown | 357(14.86%) | 326(23.01%) |  |  | 126(11.81%) | 129(12.09%) |  |
| Marital status |  |  | 0.023 |  |  |  | 1.000 |
| Married | 1558(64.86%) | 978(69.02%) |  |  | 766(71.79%) | 766(71.79%) |  |
| Single | 769(32.02%) | 394(27.81%) |  |  | 286(26.81%) | 286(26.81%) |  |
| Unknown | 75(3.12%) | 45(3.17%) |  |  | 15(1.40%) | 15(1.40%) |  |
| Race |  |  | 0.871 |  |  |  | 1.000 |
| White | 1995(83.06%) | 1174(82.85%) |  |  | 888(83.22%) | 888(83.22%) |  |
| Others | 407(16.94%) | 243(17.15%) |  |  | 179(16.78%) | 179(16.78%) |  |
| Sex |  |  | 0.804 |  |  |  | 0.633 |
| Male | 1144(47.63%) | 669(47.21%) |  |  | 508(47.61%) | 497(46.58%) |  |
| Female | 1258(52.37%) | 748(52.79%) |  |  | 559(52.39%) | 570(53.42%) |  |
| Tumor site |  |  | 0.004 |  |  |  | 0.869 |
| Pancreas Head | 1792(74.60%) | 1115(78.69%) |  |  | 865(81.07%) | 862(80.79%) |  |
| Pancreas Body Tail and other | 610(25.40%) | 302(21.31%) |  |  | 202(18.93%) | 205(19.21%) |  |
| Grade |  |  | <0.001 |  |  |  | 0.989 |
| I | 300(12.49%) | 131(9.24%) |  |  | 82(7.69%) | 78(7.31%) |  |
| II | 1044(43.46%) | 715(50.46%) |  |  | 526(49.30%) | 526(49.30%) |  |
| III/IV | 903(37.59%) | 502(35.43%) |  |  | 412(38.61%) | 416(38.99%) |  |
| Unknown | 155(6.46%) | 69(4.87%) |  |  | 47(4.40%) | 47(4.40%) |  |
| T stage |  |  | 0.010 |  |  |  | 0.992 |
| T1 | 288(11.99%) | 156(11.01%) |  |  | 100(9.37%) | 101(9.47%) |  |
| T2 | 1284(53.46%) | 829(58.50%) |  |  | 667(62.52%) | 664(62.23%) |  |
| T3 | 659(27.44%) | 328(23.15%) |  |  | 248(23.24%) | 247(23.15%) |  |
| T4 | 171(7.11%) | 104(7.34%) |  |  | 52(4.87%) | 55(5.15%) |  |
| N stage |  |  | 0.637 |  |  |  | 0.984 |
| N0 | 66(2.75%) | 32(2.26%) |  |  | 18(1.69%) | 19(1.78%) |  |
| N1 | 1459(60.74%) | 870(61.40%) |  |  | 656(61.48%) | 657(61.57%) |  |
| N2 | 877(36.51%) | 515(36.34%) |  |  | 393(36.83%) | 391(36.65%) |  |
| Chemotherapy |  |  | <0.001 |  |  |  | 1.000 |
| Yes | 1456(60.62%) | 1339(94.50%) |  |  | 1006(94.28%) | 1006(94.28%) |  |
| No/Unknown | 946(39.38%) | 78(5.50%) |  |  | 61(5.72%) | 61(5.72%) |  |
| RNE |  |  | 0.019 |  |  |  | 0.367 |
| <15 | 955(39.76%) | 629(44.39%) |  |  | 413(38.71%) | 413(38.71%) |  |
| ≥15 | 1429(59.49%) | 779(54.98%) |  |  | 654(61.29%) | 652(61.11%) |  |
| Unknown | 18(0.75%) | 9(0.63%) |  |  | 0 | 2(0.18%) |  |

Abbreviations PSM: Propensity score matching; RNE: Regional nodes examined
