# Supplementary material for: The Survival Effect of Radiotherapy on Stage IIB/III Pancreatic Cancer Undergone Surgery in Different Age and Tumor Site Groups: A Propensity Scores Matching Analysis Based on SEER Database
Source: Front Oncol. 2022 Jan 31;12:799930. doi: 10.3389/fonc.2022.799930 (PMC8841859; doi:10.3389/fonc.2022.799930)
Supplement: Supplementary file 5 [file Table_5.docx]

Supplementary Table 5. Features of elderly patients in the non-radiotherapy group and the neoadjuvant radiotherapy group before and after PSM.

| Characteristics | Before PSM | | |  | After PSM | | |
| --- | --- | --- | --- | --- | --- | --- | --- |
|  | Non-radiotherapy | Neoadjuvant radiotherapy | P |  | Non-radiotherapy | Neoadjuvant radiotherapy | P |
| Insurance Recode |  |  | 0.060 |  |  |  | 0.766 |
| Insured | 2802(84.27%) | 86(91.49%) |  |  | 79(94.05%) | 77(91.67%) |  |
| No/unknown | 523(15.73%) | 8(8.51%) |  |  | 5(5.95%) | 7(8.33%) |  |
| Marital status |  |  | 0.418 |  |  |  | 0.627 |
| Married | 1975(59.40%) | 62(65.96%) |  |  | 53(63.10%) | 57(67.86%) |  |
| Single | 1247(37.50%) | 29(30.85%) |  |  | 30(35.71%) | 25(29.76%) |  |
| Unknown | 103(3.10%) | 3(3.19%) |  |  | 1(1.19%) | 2(2.38%) |  |
| Race |  |  | 0.658 |  |  |  | 0.698 |
| White | 2842(85.47%) | 79(84.04%) |  |  | 66(78.57%) | 69(82.14%) |  |
| Others | 483(14.53%) | 15(15.96%) |  |  | 18(21.43%) | 15(17.86%) |  |
| Sex |  |  | 0.917 |  |  |  | 1.000 |
| Male | 1777(53.44%) | 51(54.26%) |  |  | 47(55.95%) | 47(55.95%) |  |
| Female | 1548(46.56%) | 43(45.74%) |  |  | 37(44.05%) | 37(44.05%) |  |
| Tumor site |  |  | 0.637 |  |  |  | 1.000 |
| Pancreas Head | 2441(73.41%) | 67(71.28%) |  |  | 61(72.62%) | 61(72.62%) |  |
| Pancreas Body Tail and other | 884(26.59%) | 27(28.72%) |  |  | 23(27.38%) | 23(27.38%) |  |
| Grade |  |  | <0.001 |  |  |  | 0.818 |
| I | 362(10.89%) | 6(6.38%) |  |  | 4(4.76%) | 5(5.95%) |  |
| II | 1529(45.98%) | 28(29.79%) |  |  | 24(28.57%) | 28(33.33%) |  |
| III/IV | 1243(37.38%) | 24(25.53%) |  |  | 27(32.14%) | 22(26.19%) |  |
| Unknown | 191(5.75%) | 36(38.30%) |  |  | 29(34.53%) | 29(34.53%) |  |
| T stage |  |  | <0.001 |  |  |  | 0.086 |
| T1 | 388(11.67%) | 2(2.13%) |  |  | 7(8.33%) | 2(2.38%) |  |
| T2 | 1892(56.90%) | 35(37.23%) |  |  | 26(30.96%) | 35(41.67%) |  |
| T3 | 839(25.23%) | 15(15.96%) |  |  | 24(28.57%) | 15(17.86%) |  |
| T4 | 206(6.20%) | 42(44.68%) |  |  | 27(32.14%) | 32(38.09%) |  |
| N stage |  |  | <0.001 |  |  |  | 0.621 |
| N0 | 82(2.47%) | 29(30.85%) |  |  | 16(19.05%) | 20(23.81%) |  |
| N1 | 2125(63.91%) | 53(56.38%) |  |  | 58(69.05%) | 52(61.90%) |  |
| N2 | 1118(33.62%) | 12(12.77%) |  |  | 10(11.90%) | 12(14.29%) |  |
| Chemotherapy |  |  | <0.001 |  |  |  | 0.497 |
| Yes | 1599(48.09%) | 94(100%) |  |  | 82(97.62%) | 84(100%) |  |
| No/Unknown | 1726(51.91%) | 0 |  |  | 2(2.38%) | 0 |  |
| RNE |  |  | 0.876 |  |  |  | 0.821 |
| <15 | 1573(47.31%) | 46(48.94%) |  |  | 42(50.00%) | 41(48.81%) |  |
| ≥15 | 1728(51.97%) | 47(50.00%) |  |  | 40(47.62%) | 42(50.00%) |  |
| Unknown | 24(0.72%) | 1(1.06%) |  |  | 2(2.38%) | 1(1.19%) |  |

Abbreviations PSM: Propensity score matching; RNE: Regional nodes examined
