# Supplementary material for: The Survival Effect of Radiotherapy on Stage IIB/III Pancreatic Cancer Undergone Surgery in Different Age and Tumor Site Groups: A Propensity Scores Matching Analysis Based on SEER Database
Source: Front Oncol. 2022 Jan 31;12:799930. doi: 10.3389/fonc.2022.799930 (PMC8841859; doi:10.3389/fonc.2022.799930)
Supplement: Supplementary file 6 [file Table_6.docx]

Supplementary Table 6. Features of elderly patients in the non-radiotherapy group and the adjuvant radiotherapy group before and after PSM.

| Characteristics | Before PSM | | |  | After PSM | | |
| --- | --- | --- | --- | --- | --- | --- | --- |
|  | Non-radiotherapy | Adjuvant radiotherapy | P |  | Non-radiotherapy | Adjuvant radiotherapy | P |
| Insurance Recode |  |  | <0.001 |  |  |  | 1.000 |
| Insured | 2802(84.27%) | 896(78.32%) |  |  | 855(85.84%) | 855(85.84%) |  |
| No/unknown | 523(15.73%) | 248(21.68%) |  |  | 141(14.16%) | 141(14.16%) |  |
| Marital status |  |  | 0.003 |  |  |  | 0.563 |
| Married | 1975(59.40%) | 744(65.03%) |  |  | 624(62.65%) | 647(64.96%) |  |
| Single | 1247(37.50%) | 365(31.91%) |  |  | 341(34.24%) | 320(32.13%) |  |
| Unknown | 103(3.10%) | 35(3.06%) |  |  | 31(3.11%) | 29(2.91%) |  |
| Race |  |  | 0.504 |  |  |  | 0.341 |
| White | 2842(85.47%) | 987(86.28%) |  |  | 843(84.64%) | 858(86.14%) |  |
| Others | 483(14.53%) | 157(13.72%) |  |  | 153(15.36%) | 138(13.86%) |  |
| Sex |  |  | 0.003 |  |  |  | 0.754 |
| Male | 1777(53.44%) | 554(48.43%) |  |  | 503(50.50%) | 496(49.80%) |  |
| Female | 1548(46.56%) | 590(51.57%) |  |  | 493(49.50%) | 500(50.20%) |  |
| Tumor site |  |  | 0.144 |  |  |  | 1.000 |
| Pancreas Head | 2441(73.41%) | 865(75.61%) |  |  | 775(77.81%) | 775(77.81%) |  |
| Pancreas Body Tail and other | 884(26.59%) | 279(24.39%) |  |  | 221(22.19%) | 221(22.19%) |  |
| Grade |  |  | 0.179 |  |  |  | 1.000 |
| I | 362(10.89%) | 102(8.92%) |  |  | 84(8.43%) | 84(8.43%) |  |
| II | 1529(45.98%) | 558(48.78%) |  |  | 488(49.00%) | 488(49.00%) |  |
| III/IV | 1243(37.38%) | 423(36.97%) |  |  | 379(38.05%) | 379(38.05%) |  |
| Unknown | 191(5.75%) | 61(5.33%) |  |  | 45(4.52%) | 45(4.52%) |  |
| T stage |  |  | 0.560 |  |  |  | 1.000 |
| T1 | 388(11.67%) | 130(11.36%) |  |  | 104(10.44%) | 104(10.44%) |  |
| T2 | 1892(56.90%) | 662(57.87%) |  |  | 618(62.05%) | 618(62.05%) |  |
| T3 | 839(25.23%) | 271(23.69%) |  |  | 226(22.69%) | 226(22.69%) |  |
| T4 | 206(6.20%) | 81(7.08%) |  |  | 48(4.82%) | 48(4.82%) |  |
| N stage |  |  | 0.333 |  |  |  | 1.000 |
| N0 | 82(2.47%) | 36(30.85%) |  |  | 19(1.91%) | 19(1.91%) |  |
| N1 | 2125(63.91%) | 711(56.38%) |  |  | 634(63.65%) | 634(63.65%) |  |
| N2 | 1118(33.62%) | 397(12.77%) |  |  | 343(34.44%) | 343(34.44%) |  |
| Chemotherapy |  |  | <0.001 |  |  |  | 1.000 |
| Yes | 1599(48.09%) | 1059(92.57%) |  |  | 913(91.67%) | 913(91.67%) |  |
| No/Unknown | 1726(51.91%) | 85(7.43%) |  |  | 83(8.33%) | 83(8.33%) |  |
| RNE |  |  | 0.527 |  |  |  | 1.000 |
| <15 | 1573(47.31%) | 525(45.89%) |  |  | 439(44.08%) | 439(44.08%) |  |
| ≥15 | 1728(51.97%) | 613(53.58%) |  |  | 557(55.92%) | 557(55.92%) |  |
| Unknown | 24(0.72%) | 6(0.53%) |  |  | 0 | 0 |  |

Abbreviations PSM: Propensity score matching; RNE: Regional nodes examined
