# Supplementary material for: The Survival Effect of Radiotherapy on Stage IIB/III Pancreatic Cancer Undergone Surgery in Different Age and Tumor Site Groups: A Propensity Scores Matching Analysis Based on SEER Database
Source: Front Oncol. 2022 Jan 31;12:799930. doi: 10.3389/fonc.2022.799930 (PMC8841859; doi:10.3389/fonc.2022.799930)
Supplement: Supplementary file 7 [file Table_7.docx]

Supplementary Table 7. Features of patients with pancreatic head cancer in the non-radiotherapy group and the neoadjuvant radiotherapy group before and after PSM.

| Characteristics | Before PSM | | |  | After PSM | | |
| --- | --- | --- | --- | --- | --- | --- | --- |
|  | Non-radiotherapy | Neoadjuvant radiotherapy | P |  | Non-radiotherapy | Neoadjuvant radiotherapy | P |
| Insurance Recode |  |  | 0.039 |  |  |  | 1.000 |
| Insured | 4649(82.87%) | 242(87.68%) |  |  | 178(90.36%) | 178(90.36%) |  |
| No/unknown | 961(17.13%) | 34(12.32%) |  |  | 19(9.64%) | 19(9.64%) |  |
| Marital status |  |  | 0.054 |  |  |  | 0.259 |
| Married | 3445(61.41%) | 185(67.03%) |  |  | 130(65.99%) | 142(72.08%) |  |
| Single | 1985(35.38%) | 79(28.62%) |  |  | 60(30.46%) | 52(26.40%) |  |
| Unknown | 180(3.21%) | 12(4.35%) |  |  | 7(3.55%) | 3(1.52%) |  |
| Age |  |  | <0.001 |  |  |  | 0.484 |
| < 60 | 1377(24.55%) | 103(37.32%) |  |  | 70(35.53%) | 64(32.49%) |  |
| 60-69 | 1792(31.94%) | 106(38.41%) |  |  | 86(43.66%) | 82(41.62%) |  |
| ≥70 | 2441(43.51%) | 67(24.27%) |  |  | 41(20.81%) | 51(25.89%) |  |
| Race |  |  | 0.006 |  |  |  | 0.122 |
| White | 4656(82.99%) | 246(89.13%) |  |  | 161(81.73%) | 173(87.81%) |  |
| Others | 954(17.01%) | 30(10.87%) |  |  | 36(18.27%) | 24(12.18%) |  |
| Sex |  |  | 0.724 |  |  |  | 0.481 |
| Male | 2744(48.91%) | 138(50.00%) |  |  | 104(52.79%) | 97(49.24%) |  |
| Female | 2866(51.09%) | 138(50.00%) |  |  | 93(47.21%) | 100(50.76%) |  |
| Grade |  |  | <0.001 |  |  |  | 1.000 |
| I | 633(11.28%) | 19(6.88%) |  |  | 12(6.09%) | 12(6.09%) |  |
| II | 2504(44.63%) | 86(31.16%) |  |  | 75(38.07%) | 75(38.07%) |  |
| III/IV | 2110(37.62%) | 72(26.09%) |  |  | 51(25.89%) | 51(25.89%) |  |
| Unknown | 363(6.47%) | 99(35.87%) |  |  | 59(29.95%) | 59(29.95%) |  |
| T stage |  |  | <0.001 |  |  |  | 0.092 |
| T1 | 725(12.92%) | 10(3.62%) |  |  | 21(10.66%) | 8(4.06%) |  |
| T2 | 3380(60.25%) | 119(43.12%) |  |  | 93(47.21%) | 103(52.28%) |  |
| T3 | 1169(20.84%) | 52(18.84%) |  |  | 38(19.29%) | 41(20.82%) |  |
| T4 | 336(5.99%) | 95(34.42%) |  |  | 45(22.84%) | 45(22.84%) |  |
| N stage |  |  | <0.001 |  |  |  | 1.000 |
| N0 | 110(1.96%) | 70(25.36%) |  |  | 28(14.21%) | 28(14.21%) |  |
| N1 | 3349(59.70%) | 173(62.68%) |  |  | 139(70.56%) | 139(70.56%) |  |
| N2 | 2151(38.34%) | 33(11.96%) |  |  | 30(15.23%) | 30(15.23%) |  |
| Chemotherapy |  |  | <0.001 |  |  |  | 1.000 |
| Yes | 3195(56.95%) | 275(99.64%) |  |  | 197(100%) | 197(100%) |  |
| No/Unknown | 2415(43.05%) | 1(0.36%) |  |  | 0 | 0 |  |
| RNE |  |  | <0.001 |  |  |  | 1.000 |
| <15 | 2303(41.05%) | 138(50.00%) |  |  | 90(45.69%) | 90(45.69%) |  |
| ≥15 | 3267(58.24%) | 131(47.46%) |  |  | 106(53.81%) | 106(53.81%) |  |
| Unknown | 40(0.71%) | 7(2.54%) |  |  | 1(0.50%) | 1(0.50%) |  |

Abbreviations PSM: Propensity score matching; RNE: Regional nodes examined
