# Supplementary material for: The Survival Effect of Radiotherapy on Stage IIB/III Pancreatic Cancer Undergone Surgery in Different Age and Tumor Site Groups: A Propensity Scores Matching Analysis Based on SEER Database
Source: Front Oncol. 2022 Jan 31;12:799930. doi: 10.3389/fonc.2022.799930 (PMC8841859; doi:10.3389/fonc.2022.799930)
Supplement: Supplementary file 8 [file Table_8.docx]

Supplementary Table 8. Features of patients with pancreatic head cancer in the non-radiotherapy group and the adjuvant radiotherapy group before and after PSM.

| Characteristics | Before PSM | | |  | After PSM | | |
| --- | --- | --- | --- | --- | --- | --- | --- |
|  | Non-radiotherapy | Adjuvant radiotherapy | P |  | Non-radiotherapy | Adjuvant radiotherapy | P |
| Insurance Recode |  |  | <0.001 |  |  |  | 1.000 |
| Insured | 4649(82.87%) | 2193(75.39%) |  |  | 1780(85.78%) | 1780(85.78%) |  |
| No/unknown | 961(17.13%) | 716(24.61%) |  |  | 295(14.22%) | 295(14.22%) |  |
| Marital status |  |  | <0.001 |  |  |  | 0.988 |
| Married | 3445(61.41%) | 1934(66.48%) |  |  | 1444(69.59%) | 1442(69.49%) |  |
| Single | 1985(35.38%) | 888(30.53%) |  |  | 608(29.30%) | 609(29.35%) |  |
| Unknown | 180(3.21%) | 87(2.99%) |  |  | 23(1.11%) | 24(1.16%) |  |
| Age |  |  | <0.001 |  |  |  | 0.981 |
| < 60 | 1377(24.55%) | 929(31.94%) |  |  | 612(29.49%) | 607(29.25%) |  |
| 60-69 | 1792(31.94%) | 1115(38.33%) |  |  | 788(37.98%) | 788(37.98%) |  |
| ≥70 | 2441(43.51%) | 865(29.73%) |  |  | 675(32.53%) | 680(32.77%) |  |
| Race |  |  | 0.405 |  |  |  | 0.475 |
| White | 4656(82.99%) | 2435(83.71%) |  |  | 1728(83.28%) | 1745(84.10%) |  |
| Others | 954(17.01%) | 474(16.29%) |  |  | 347(16.72%) | 330(15.90%) |  |
| Sex |  |  | 0.033 |  |  |  | 0.827 |
| Male | 2744(48.91%) | 1352(46.48%) |  |  | 957(46.12%) | 964(46.46%) |  |
| Female | 2866(51.09%) | 1557(53.52%) |  |  | 1118(53.88%) | 1111(53.54%) |  |
| Grade |  |  | <0.001 |  |  |  | 0.929 |
| I | 633(11.28%) | 260(8.94%) |  |  | 135(6.51%) | 134(6.46%) |  |
| II | 2504(44.63%) | 1449(49.81%) |  |  | 1029(49.59%) | 1039(50.07%) |  |
| III/IV | 2110(37.62%) | 1057(36.34%) |  |  | 827(39.86%) | 826(39.81%) |  |
| Unknown | 363(6.47%) | 143(4.91%) |  |  | 84(4.04%) | 76(3.66%) |  |
| T stage |  |  | 0.202 |  |  |  | 0.989 |
| T1 | 725(12.92%) | 390(13.41%) |  |  | 231(11.13%) | 225(10.84%) |  |
| T2 | 3380(60.25%) | 1751(60.19%) |  |  | 1395(67.23%) | 1397(67.33%) |  |
| T3 | 1169(20.84%) | 567(19.49%) |  |  | 381(18.36%) | 386(18.60%) |  |
| T4 | 336(5.99%) | 201(6.91%) |  |  | 68(3.28%) | 67(3.23%) |  |
| N stage |  |  | 0.899 |  |  |  | 0.971 |
| N0 | 110(1.96%) | 61(2.10%) |  |  | 17(0.82%) | 18(0.87%) |  |
| N1 | 3349(59.70%) | 1740(59.81%) |  |  | 1254(60.43%) | 1248(60.14%) |  |
| N2 | 2151(38.34%) | 1108(38.09%) |  |  | 804(38.75%) | 809(38.99%) |  |
| Chemotherapy |  |  | <0.001 |  |  |  | 1.000 |
| Yes | 3195(56.95%) | 2762(94.95%) |  |  | 1961(94.51%) | 1961(94.51%) |  |
| No/Unknown | 2415(43.05%) | 147(5.05%) |  |  | 114(5.49%) | 114(5.49%) |  |
| RNE |  |  | 0.120 |  |  |  | 0.905 |
| <15 | 2303(41.05%) | 1257(43.21%) |  |  | 792(38.17%) | 792(38.17%) |  |
| ≥15 | 3267(58.24%) | 1636(56.24%) |  |  | 1280(61.69%) | 1281(61.73%) |  |
| Unknown | 40(0.71%) | 16(0.55%) |  |  | 3(0.14%) | 2(0.10%) |  |

Abbreviations PSM: Propensity score matching; RNE: Regional nodes examined
