# Supplementary material for: The Survival Effect of Radiotherapy on Stage IIB/III Pancreatic Cancer Undergone Surgery in Different Age and Tumor Site Groups: A Propensity Scores Matching Analysis Based on SEER Database
Source: Front Oncol. 2022 Jan 31;12:799930. doi: 10.3389/fonc.2022.799930 (PMC8841859; doi:10.3389/fonc.2022.799930)
Supplement: Supplementary file 9 [file Table_9.docx]

Supplementary Table 9. Features of PDAC patients at other sites in the non-radiotherapy group and the neoadjuvant radiotherapy group before and after PSM.

| Characteristics | Before PSM | | |  | After PSM | | |
| --- | --- | --- | --- | --- | --- | --- | --- |
|  | Non-radiotherapy | Neoadjuvant radiotherapy | P |  | Non-radiotherapy | Neoadjuvant radiotherapy | P |
| Insurance Recode |  |  | 0.677 |  |  |  | 0.521 |
| Insured | 1793(84.38%) | 88(86.27%) |  |  | 65(81.25%) | 69(86.25%) |  |
| No/unknown | 332(15.62%) | 14(13.73%) |  |  | 15(18.75%) | 11(13.75%) |  |
| Marital status |  |  | <0.001 |  |  |  | 0.062 |
| Married | 1307(61.51%) | 88(86.27%) |  |  | 56(70.00%) | 67(83.75%) |  |
| Single | 740(34.82%) | 12(11.77%) |  |  | 23(28.75%) | 11(13.75%) |  |
| Unknown | 78(3.67%) | 2(1.96%) |  |  | 1(1.25%) | 2(2.50%) |  |
| Age |  |  | 0.006 |  |  |  | 0.196 |
| < 60 | 631(29.69%) | 34(33.33%) |  |  | 36(45.00%) | 25(31.25%) |  |
| 60-69 | 610(28.71%) | 41(40.20%) |  |  | 29(36.25%) | 35(43.75%) |  |
| ≥70 | 884(41.60%) | 27(26.47%) |  |  | 15(18.75%) | 20(25.00%) |  |
| Race |  |  | 0.606 |  |  |  | 0.563 |
| White | 1727(81.27%) | 81(79.41%) |  |  | 65(81.25%) | 61(76.25%) |  |
| Others | 398(18.73%) | 21(20.59%) |  |  | 15(18.75%) | 19(23.75%) |  |
| Sex |  |  | 0.187 |  |  |  | 0.268 |
| Male | 1069(50.31%) | 44(43.14%) |  |  | 44(55.00%) | 36(45.00%) |  |
| Female | 1056(49.69%) | 58(56.86%) |  |  | 36(45.00%) | 44(55.00%) |  |
| Grade |  |  | <0.001 |  |  |  | 0.592 |
| I | 405(19.06%) | 12(11.76%) |  |  | 10(12.50%) | 11(13.75%) |  |
| II | 890(41.88%) | 33(32.35%) |  |  | 25(31.25%) | 27(33.75%) |  |
| III/IV | 653(30.73%) | 18(17.65%) |  |  | 20(25.00%) | 13(16.25%) |  |
| Unknown | 177(8.33%) | 39(38.24%) |  |  | 25(31.25%) | 29(36.25%) |  |
| T stage |  |  | <0.001 |  |  |  | 0.068 |
| T1 | 203(9.55%) | 0 |  |  | 4(5.00%) | 0 |  |
| T2 | 847(39.86%) | 17(16.67%) |  |  | 9(11.25%) | 17(21.25%) |  |
| T3 | 888(41.79%) | 17(16.67%) |  |  | 22(27.50%) | 17(21.25%) |  |
| T4 | 187(8.80%) | 68(66.66%) |  |  | 45(56.25%) | 46(57.50%) |  |
| N stage |  |  | <0.001 |  |  |  | 0.351 |
| N0 | 92(4.33%) | 47(46.08%) |  |  | 33(41.25%) | 30(37.50%) |  |
| N1 | 1443(67.91%) | 46(45.10%) |  |  | 33(41.25%) | 41(51.25%) |  |
| N2 | 590(27.76%) | 9(8.82%) |  |  | 14(17.50%) | 9(11.25%) |  |
| Chemotherapy |  |  | <0.001 |  |  |  | 1.000 |
| Yes | 1002(47.15%) | 101(99.02%) |  |  | 78(97.50%) | 79(98.75%) |  |
| No/Unknown | 1123(52.85%) | 1(0.98%) |  |  | 2(2.50%) | 1(1.25%) |  |
| RNE |  |  | 0.008 |  |  |  | 0.765 |
| <15 | 1116(52.52%) | 60(58.82%) |  |  | 41(51.25%) | 44(55.00%) |  |
| ≥15 | 996(46.87%) | 39(38.24%) |  |  | 37(46.25%) | 33(41.25%) |  |
| Unknown | 13(0.61%) | 3(2.94%) |  |  | 2(2.50%) | 3(3.75%) |  |

Abbreviations PSM: Propensity score matching; RNE: Regional nodes examined
