# Supplementary material for: The Survival Effect of Radiotherapy on Stage IIB/III Pancreatic Cancer Undergone Surgery in Different Age and Tumor Site Groups: A Propensity Scores Matching Analysis Based on SEER Database
Source: Front Oncol. 2022 Jan 31;12:799930. doi: 10.3389/fonc.2022.799930 (PMC8841859; doi:10.3389/fonc.2022.799930)
Supplement: Supplementary file 10 [file Table_10.docx]

Supplementary Table 10. Features of PDAC patients at other sites in the non-radiotherapy group and the adjuvant radiotherapy group before and after PSM.

| Characteristics | Before PSM | | |  | After PSM | | |
| --- | --- | --- | --- | --- | --- | --- | --- |
|  | Non-radiotherapy | Adjuvant radiotherapy | P |  | Non-radiotherapy | Adjuvant radiotherapy | P |
| Insurance Recode |  |  | <0.001 |  |  |  | 1.000 |
| Insured | 1793(84.38%) | 657(77.94%) |  |  | 585(86.41%) | 585(86.41%) |  |
| No/unknown | 332(15.62%) | 186(22.06%) |  |  | 92(13.59%) | 92(13.59%) |  |
| Marital status |  |  | 0.031 |  |  |  | 0.781 |
| Married | 1307(61.51%) | 562(66.67%) |  |  | 457(67.50%) | 462(68.24%) |  |
| Single | 740(34.82%) | 253(30.01%) |  |  | 207(30.58%) | 199(29.40%) |  |
| Unknown | 78(3.67%) | 28(3.32%) |  |  | 13(1.92%) | 16(2.36%) |  |
| Age |  |  | <0.001 |  |  |  | 0.772 |
| < 60 | 631(29.69%) | 262(31.08%) |  |  | 213(31.46%) | 203(29.99%) |  |
| 60-69 | 610(28.71%) | 302(35.82%) |  |  | 249(36.78%) | 248(36.63%) |  |
| ≥70 | 884(41.60%) | 279(33.10%) |  |  | 215(31.76%) | 226(33.38%) |  |
| Race |  |  | 0.453 |  |  |  | 0.171 |
| White | 1727(81.27%) | 675(80.07%) |  |  | 554(81.83%) | 534(78.88%) |  |
| Others | 398(18.73%) | 168(19.93%) |  |  | 123(18.17%) | 143(21.12%) |  |
| Sex |  |  | 0.266 |  |  |  | 0.254 |
| Male | 1069(50.31%) | 405(48.04%) |  |  | 355(52.44%) | 334(49.34%) |  |
| Female | 1056(49.69%) | 438(51.96%) |  |  | 322(47.56%) | 343(50.66%) |  |
| Grade |  |  | <0.001 |  |  |  | 0.218 |
| I | 405(19.06%) | 81(9.61%) |  |  | 55(8.12%) | 60(8.86%) |  |
| II | 890(41.88%) | 404(47.92%) |  |  | 323(47.71%) | 333(49.19%) |  |
| III/IV | 653(30.73%) | 309(36.65%) |  |  | 250(36.93%) | 253(37.37%) |  |
| Unknown | 177(8.33%) | 49(5.81%) |  |  | 49(7.24%) | 31(4.58%) |  |
| T stage |  |  | 0.074 |  |  |  | 0.750 |
| T1 | 203(9.55%) | 65(7.71%) |  |  | 49(7.24%) | 46(6.79%) |  |
| T2 | 847(39.86%) | 370(43.89%) |  |  | 327(48.30%) | 325(48.01%) |  |
| T3 | 888(41.79%) | 325(38.55%) |  |  | 268(39.59%) | 264(39.00%) |  |
| T4 | 187(8.80%) | 83(9.85%) |  |  | 33(4.87%) | 42(6.20%) |  |
| N stage |  |  | 0.728 |  |  |  | 0.834 |
| N0 | 92(4.33%) | 34(4.03%) |  |  | 14(2.07%) | 17(2.51%) |  |
| N1 | 1443(67.91%) | 585(69.40%) |  |  | 479(70.75%) | 481(71.05%) |  |
| N2 | 590(27.76%) | 224(26.57%) |  |  | 184(27.18%) | 179(26.44%) |  |
| Chemotherapy |  |  | <0.001 |  |  |  | 1.000 |
| Yes | 1002(47.15%) | 777(92.17%) |  |  | 615(90.84%) | 615(90.84%) |  |
| No/Unknown | 1123(52.85%) | 66(7.83%) |  |  | 62(9.16%) | 62(9.16%) |  |
| RNE |  |  | 0.177 |  |  |  | 0.629 |
| <15 | 1116(52.52%) | 412(48.87%) |  |  | 318(46.97%) | 329(48.60%) |  |
| ≥15 | 996(46.87%) | 424(50.30%) |  |  | 356(52.59%) | 343(50.66%) |  |
| Unknown | 13(0.61%) | 7(0.83%) |  |  | 3(0.44%) | 5(0.74%) |  |

Abbreviations PSM: Propensity score matching; RNE: Regional nodes examined
